# Nuck's Canal Cyst Associated With Endometriosis: A Case Report

**DOI:** 10.7759/cureus.84231

**Published:** 2025-05-16

**Authors:** Catalina Ortiz-Monasterio, Piero Carvallo-Maiocco, Martín Vega-de Jesús, Paulina P Rábago-Sánchez, César O Decanini-Terán

**Affiliations:** 1 Surgery Department, ABC Medical Center, Mexico City, MEX

**Keywords:** endometriosis, inguinal hernia, inguinal plasty, nuck's canal, nuck's cyst

## Abstract

Nuck's canal is the incomplete obliteration of the processus vaginalis, and it is a rare condition that should consider inguinodynia and an increased volume in the inguinal region as a differential diagnosis. Endometriosis is a systemic disease that affects young women. This condition has many clinical presentations, and it is exceptional to find it associated with a Nuck's canal cyst. We present the case of a 20-year-old woman who presented with right inguinal pain associated with increased volume. An inguinal ultrasound and abdominopelvic computed tomography (CT) were performed, and a right Nuck's canal cyst was diagnosed. The condition was managed surgically with resection of the Nuck's canal cyst and transabdominal preperitoneal plasty (TAPP) for inguinal hernia laparoscopic reconstruction, with a satisfactory outcome.

## Introduction

Nuck's canal refers to the incomplete obliteration of the processus vaginalis, it typically occurs in early childhood. It was first described by the Dutch surgeon and anatomist Anton Nuck in 1691 [1]. Its incidence is unknown due to the low number of reported cases, although it is known to be more common in young women and is typically treated before the age of five [2]. During embryological development, the round ligament, a structure derived from the lower portion of the gubernaculum, attaches to the uterus and the labia majora. When this pathway is incompletely obliterated in the processus vaginalis, it is known as Nuck's canal, which is associated with inguinal hernias and cysts. The clinical presentation is nonspecific, with increased volume, swelling, and pain in the inguinal region. The diagnostic approach begins with a physical examination accompanied by an inguinal ultrasound and, if necessary, can be complemented with an abdominal CT or magnetic resonance imaging (MRI) [1-3].

Endometriosis is a common condition with a reported incidence of 5-10% in the global population of fertile women, affecting 50-60% of women with pelvic pain and 50% of women with infertility [4,5]. This condition can be associated with inguinal hernias and Nuck's canal cysts, although this presentation is extremely rare.

We present a case report of a young woman with pelvic pain associated with increased volume in the right inguinal region, in whom a Nuck's cyst associated with endometriosis was confirmed by histopathological studies. The case was successfully resolved via laparoscopy, demonstrating the potential for positive outcomes in such rare and complex cases.

## Case presentation

**Figure 1:**
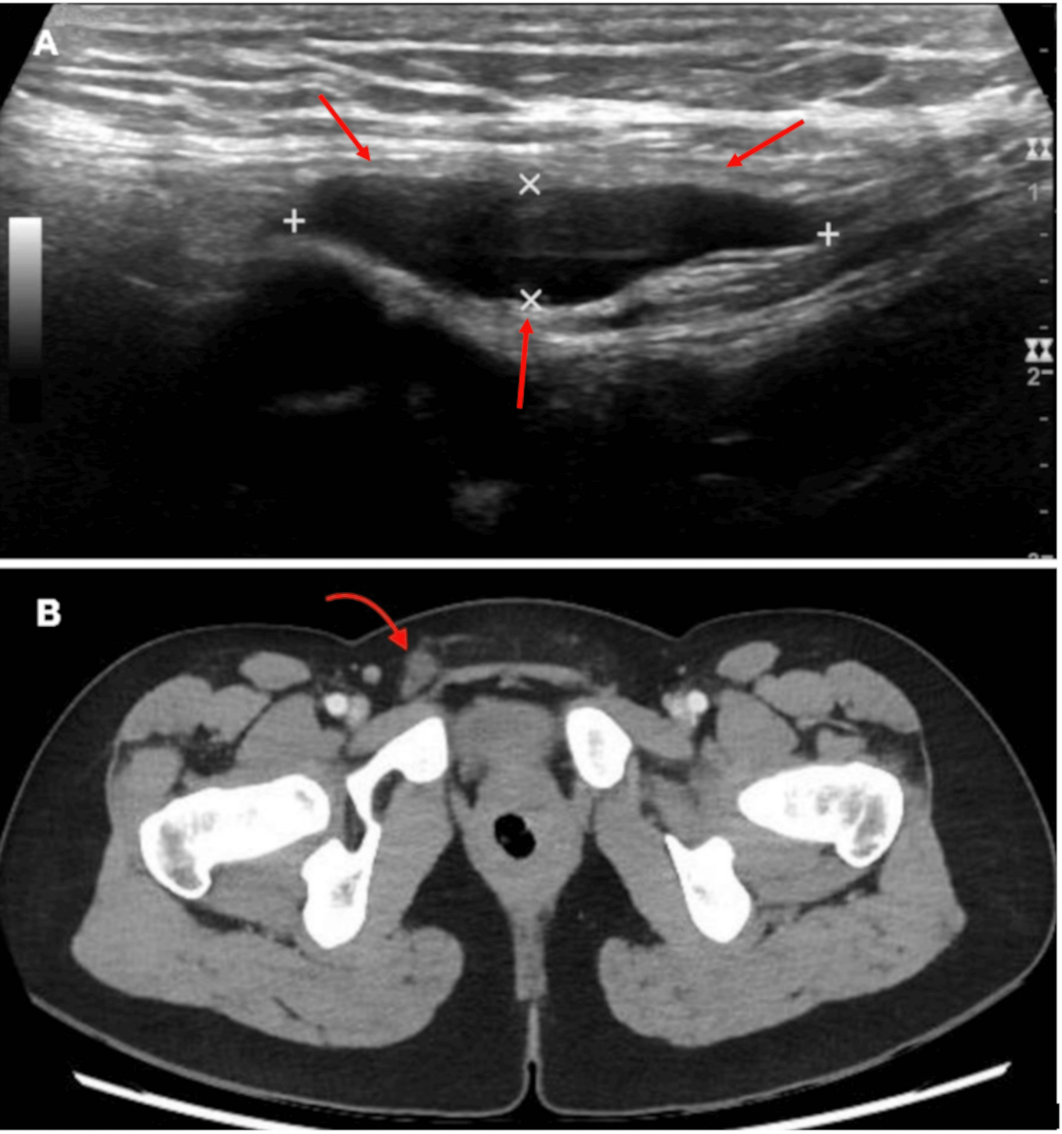
Patient imaging studies A) Ultrasound of the right inguinal region showing a cyst in the lower inguinal area measuring 2.8 x 0.6 cm, with no evidence of an abdominal wall defect. The image is consistent with a cyst of the right canal of Nuck. B) Abdominopelvic CT scan showing a rounded, fluid-filled lesion (9.6 Hounsfield units (HU)) in the right inguinal region with well-defined borders, homogeneous in appearance, measuring approximately 1 x 1.5 cm, and without communication with the peritoneal cavity (Type I). Red arrows indicate the imaging findings in each panel.

A 20-year-old nulliparous female patient with a gynecological and obstetric history of coursing with intermittent hypogastric pain that worsened during menstruation, no previous diagnosis of endometriosis was reported. With no other significant medical history presented. Referred right inguinal pain associated with a progressive increase in volume in the ipsilateral inguinal region, which worsens with the Valsalva maneuver. Physical examination revealed no defects in the superficial or deep inguinal orifices, but a mass was palpated in the right inguinal region during the Valsalva maneuver. A right inguinal ultrasound revealed a cyst in the lower inguinal region measuring 2.8 x 0.6 cm, with no evidence of an abdominal wall defect, consistent with a right Nuck's canal cyst. The diagnostic approach was complemented with an abdominopelvic CT, which reported a rounded, well-defined, homogeneous fluid-filled image of 9.6 Hounsfield units (HU) (units that represent in tomography soft tissue density) in the right inguinal region, measuring approximately 1 x 1.5 cm, with no communication with the peritoneal cavity (Type I), concluding a Type I right Nuck's canal cyst (Figure 1).

**Figure 2:**
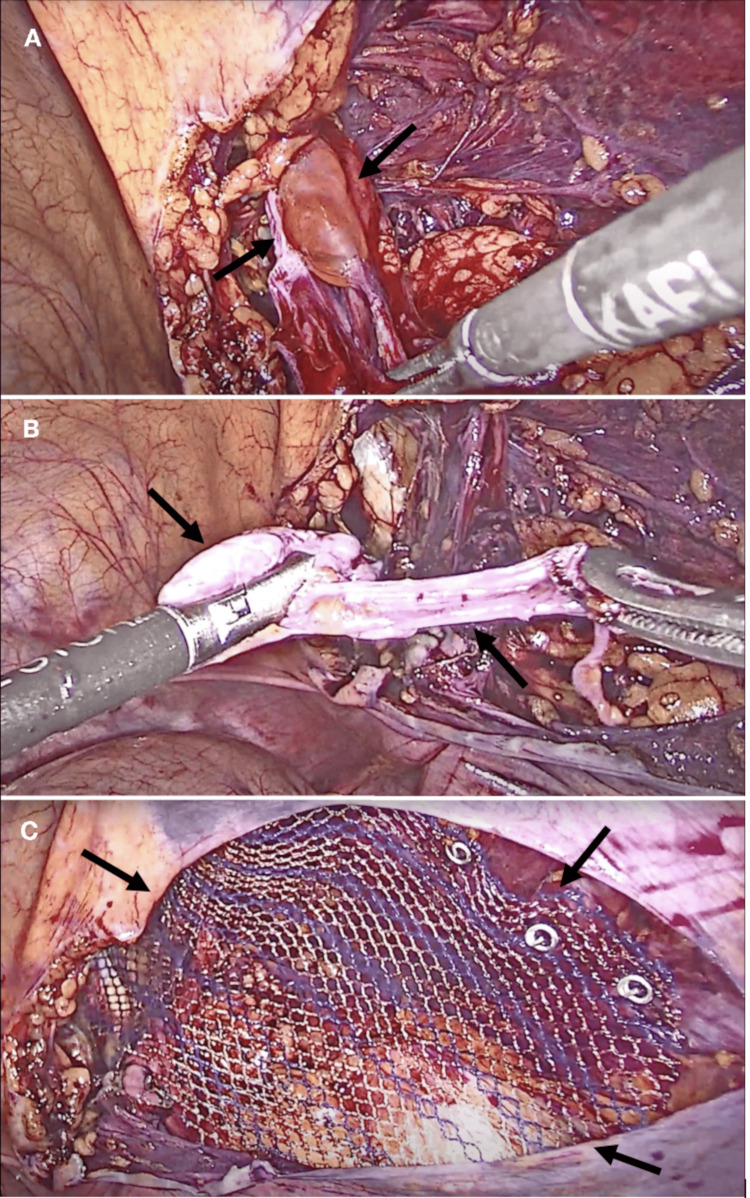
Laparoscopic intervention A) Cyst of the canal of Nuck on the right side. B) Cyst of the canal of Nuck completely resected along with its capsule. C) Preformed lightweight polypropylene mesh fixed with non-absorbable tackers in the right inguinal region, covering the right myopectineal orifice. Black arrows indicate the anatomical defects in each panel.

The patient was admitted for prompt surgical evaluation and management. Intravenous ceftriaxone was administered prior to surgery. A laparoscopic resection of the right Nuck's cyst and transabdominal preperitoneal plasty (TAPP) was performed. The peritoneal flap and inguinal structures were dissected, identifying the round ligament with a hydrocele in Nuck's canal, which was completely resected and sent for pathology. A lightweight preformed polypropylene mesh was placed and fixed with non-absorbable tackers (Figure 2). The peritoneal defect was closed with an absorbable wound closure device. The procedure lasted one hour and 15 minutes with 10 ml of blood loss. The postoperative course was uneventful, and the patient recovered without complications.

**Figure 3:**
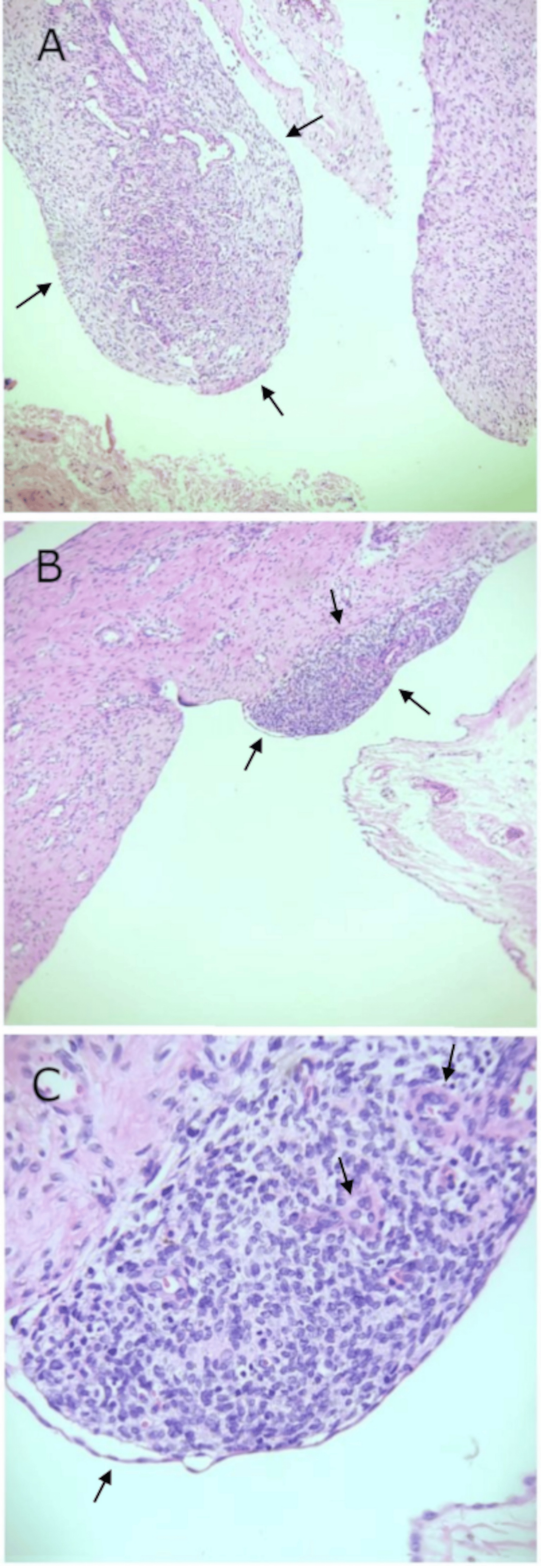
Histopathological Findings of a Canal of Nuck Cyst Associated with Endometriosis A) Cyst wall from the canal of Nuck showing dense fibrous tissue with spindle-shaped cells and scattered blood vessels, compatible with a mesothelial-lined cystic structure. H&E stain, 10x magnification. B) Proliferation of endometrial-type stroma infiltrating the fibrous wall of the cyst, further supporting the diagnosis of endometriosis associated with a canal of Nuck cyst. H&E stain, 10x magnification. C) High-power view revealing endometrial-type glands surrounded by endometrial stroma embedded within the cyst wall, consistent with ectopic endometrial tissue. H&E stain, 40x magnification. Black arrows indicate the histopathological findings in each panel.

Histopathological examination reported a benign multiloculated mesothelial cyst measuring 3.5 x 0.5 x 0.2 cm and focal endometriosis (endometrial stroma) with old hemorrhage and segmental fibrosis, confirming a Nuck's cyst (Figure 3).

## Discussion

The processus vaginalis is a tubular fold of the parietal peritoneum that extends into the inguinal canal by the seventh month of gestation and obliterates between the eighth month of gestation and the first year of life. It enters the inguinal canal through the deep orifice and positions itself anterior to the gubernaculum before shifting medially. When the canal of Nuck fails to obliterate, it may allow the passage of intra-abdominal contents along the inguinal canal, reaching the labia majora. However, if only the superior portion of the canal remains obliterated, it can lead to the development of cysts within the duct [1].

Different types of Nuck's canal cysts have been described (Table 1), including Type I, a functioning hydrocele; Type II, an encysted hydrocele; and Type III, a binocular or "hourglass" cyst.

**Table 1:** Types of Nuck's cyst. This table was based on [1,6]

Type	Characteristics	Frequency	Complications
I (Functional)	Partial failure in the obliteration of the processus vaginalis. Persistent communication with the peritoneum through the deep inguinal ring, allowing fluid passage.	Intermediate frequency	Infection, hemorrhage, inguinal hernia
II (Encysted)	Normal obliteration of the processus vaginalis with persistence of a distal segment, without communication with the peritoneal cavity.	Most frequent	Infection, hemorrhage
III (Hourglass)	The inguinal ring compresses the hydrocele, forming two cysts. The proximal cyst communicates with the peritoneal cavity, while the distal cyst does not (bilocular hydrocele).	Least frequent	Infection, hemorrhage

Endometriosis is a chronic and debilitating disease associated with pelvic pain and infertility. It can be superficial, infiltrative, or present as endometriomas [4]. The inguinal region is an exceptional site for this disease, even rarer when associated with a Nuck's cyst. However, it should be included in the differential diagnosis when obvious causes of inguinal pain have been ruled out [7,8].

In a recent systematic review by Prodromidou et al. [3], 36 cases of endometriosis associated with Nuck's cysts were found in 20 studies, with an estimated prevalence of 0.3-6%. Of these, 92% were of reproductive age, with a predominance of right inguinal cysts in over 80% of cases. In five out of 36 cases, adenocarcinoma was the most frequently encountered type among the malignant tumors. Management was surgical in all cases, ranging from simple cyst resection performing open or laparoscopic approaches to complex oncological resections with reconstruction. All patients were alive at 37 months of follow-up.

Diagnosis should be based on physical examination, as most cases reported in the literature were diagnosed during surgery. High-resolution ultrasound can complement diagnosis, which often identifies nonspecific masses. Differential diagnoses include indirect hernia, inguinal lymphadenopathy, cold abscesses, malignancy, endometriosis, Bartholin's cysts, or post-traumatic hematomas. Differential diagnosis should be made [3,9]. Routine abdominopelvic CT or MRI is not recommended, but it may be helpful to exclude the previously mentioned differential diagnoses [2].

Management is surgical, involving total cyst resection and inguinal plasty in all cases to resolve the disease and symptoms [2]. When cysts are associated with endometriosis, a multidisciplinary approach involving surgery, gynecology, imaging, and pathology services is necessary for identifying and providing the best approach for each patient [7]. The decision to perform an open or laparoscopic approach depends on the surgeon's preference and the type of cyst. Aspiration of the cyst is not a management option, as recurrence, although rare, is a late complication that can occur within the first 24 months after the disease [10,11].

## Conclusions

Nuck's canal cysts are a rare entity that should be considered as a differential diagnosis for pain and increased volume in the inguinal region by general surgeons. Endometriosis is a systemic disease that affects young women, has many clinical presentations, and is exceptionally rare when associated with a Nuck's canal cyst. The diagnostic approach is based on a detailed physical examination of the inguinal region accompanied by an ultrasound, but CT or MRI can be used if suspicion is high. Management in all cases is surgical, with cyst resection and plasty, as presented in our case. When associated with endometriosis, management should be multidisciplinary.
